# Mitral annular systolic velocity as a marker of preclinical systolic dysfunction among patients with arterial hypertension

**DOI:** 10.1186/1476-7120-10-46

**Published:** 2012-11-28

**Authors:** Ivaylo Rilkov Daskalov, Plamen Dimitrov Petrovsky, Lilia Davidkova Demirevska

**Affiliations:** 1 Department of Cardiology and Intensive Care, Cardiology Clinic, Military Medical Academy, 3 Georgi Sofiiski Blvd., Sofia, 1606, Bulgaria; 2 Department of Cardiology and Intensive Care, Unit of Functional Diagnostics of Cardiovascular System, Military Medical Academy, 3 Georgi Sofiiski Blvd., Sofia, 1606, Bulgaria

**Keywords:** Early changes, Long axis systolic function, Hypertension, Diastolic dysfunction

## Abstract

**Background:**

The aim of this study was to investigate early changes in left ventricular longitudinal systolic function in patients with hypertension (HTN) with and without concomitant diastolic dysfunction (DD) and the clinical implications of these findings.

**Method:**

We enrolled 299 patients with HTN and 297 age-matched patients with HTN and DD and compared both groups with an age-matched control group consisting of 100 healthy subjects. The long axis systolic function was investigated by determining the average peak systolic velocity of the septal and lateral mitral sites (Sm_avg_) using spectral pulsed wave tissue Doppler imaging (TDI).

**Results:**

We found a strong negative trend toward the reduction of velocity, which is dependent on the grade of HTN, on the magnitude of DD, and also on the gender and age of the subjects (r=−0.891/-0.580; p<0.0001). The data showed that the beginning and evolution of HTN are related to a slight but significant reduction in the long axis systolic function (10.2-10.0 cm/s; p<0.0001), and DD worsens this initial finding (9.8-8.8 cm/s; p<0.0001).

**Conclusion:**

The strength of the study is the analysis of incremental changes in longitudinal contraction in patients with different stage of HTN but not so many the classification of the degree of systolic dysfunction. The importance of our results lies in the fact that these initial changes in systolic contraction could be used as an early sign that should prompt optimization of the treatment of HTN.

## Background

Hypertension (HTN) is the most common cardiovascular disease and a major public health problem in both developed and developing countries [1]. Worldwide, high blood pressure is estimated to cause 7.1 million deaths every year, which represents approximately 13% of the global overall mortality. Approximately 62% of strokes and 49% of heart attacks are caused by high blood pressure [2]. HTN causes 5 million premature deaths a year worldwide. Typically, it begins without clinical symptoms, which makes early diagnosis and treatment difficult. Later in the course of the disease, left ventricular (LV) hypertrophy and DD develop, if no appropriate therapy for HTN is given in time. The most common symptoms in this stage of the disease are dyspnea and fatigue, initially on exertion and later at rest. The underlying mechanism is elevated LV filling pressure, which leads to different degrees of pulmonary congestion [3].

However, some studies suggest that minimal systolic impairment in the longitudinal contraction of the LV contribute to the above mentioned symptoms in patients with HTN especially when DD is presented [4-9]. The dilemma is how to study this early changes in longitudinal systolic function.

There is great methodological heterogeneity in determining myocardial velocities. New data have come from a recent expert consensus statement of American Society of Echocardiography (ASE) and European Association of Echocardiography (EAE) regarding currently available and evolving echocardiographic techniques that allow the quantitative assessment of cardiac mechanics via image-based analysis [10]. In summary, the consensus advocate using the well-studied spectral pulsed wave TDI. Other techniques that allow quantitative assessment of myocardial function via image-based analysis are not suitable for everyday practice because they require advanced levels of skill, lack standardization and are time-consuming. TDI has emerged as one of the most powerful prognosticators for cardiovascular disease in the realm of non-invasive cardiac imaging [11-13]. The ability to quantify long-axis function of the myocardium in a reproducible manner has also significantly refined the assessment of LV function.

The aim of this study was to investigate mitral annular systolic velocity by TDI as a marker of preclinical systolic dysfunction among patients with HTN and determine the influence of age, gender and DD.

## Methods

Single center cross-sectional analysis of 3 populations (Healthy, HTN, and DD pts.). Subjects were randomly selected between September 2007 and August 2011 from outpatient clinic and hospitalized patients at the Department of Cardiology and Intensive Care at Military Medical Academy, Sofia, Bulgaria. In this study were enrolled 299 patients with HTN without DD, 297 patients with HTN with DD and 100 aged and gender-matched healthy subjects (Table 1). All the patients (n=696) were in sinus rhythm and had a preserved LV ejection fraction (LVEF). There were no patients with signs and symptoms of coronary heart disease, diabetes mellitus or more than a mild valvular heart disease. The basal demographics and clinical parameters are presented on the Table 2. All participants signed an informed consent form. Assessment of participants was performed by experienced cardiologists using a standard protocol, including questions on medical history, family history, cardiovascular risk factors, diabetes mellitus history, alcohol intake, physical activity and drug history. A physical examination was performed, which included blood pressure, anthropometric measurements, and an electrocardiogram. The standard laboratory blood tests were performed to identify subjects with diabetes mellitus (fasting blood glucose), dyslipidemia (cholesterol, LDL, HDL, TG), anemia (Hb), significant liver (AST, ALT, GGT, bilirubin) and kidney disease (creatinine, urea). After that the eligible subjects were invited to undergo echocardiography. Echocardiography was performed in a left lateral decubitus position, with a digital commercial harmonic imaging ultrasound system, АLOKA PROSOUND SSD5500 SV, equipped with a 2.5 MHz phased-array transducer. The images were acquired during a breath hold. LV dimensions were obtained in the parasternal short axis view, and LV mass was calculated using the Devereaux formula and indexed to height to give the LV mass index (LVMI). LV hypertrophy was investigated by LVMI and the thickness of the walls. The EF was assessed using the biplane Simpson’s method and assume to be preserved when it was equal or more than 55% [14]. Left atrial volume was calculated from 3 measurements of left atrial dimensions using the formula for an ellipse and indexed to body surface area to obtain the left atrial volume index (LAVI). Diastolic function of the LV was assessed using the following indices: ratio E/A, Valsalva maneuver ΔE/A, ratio E/e′, LAVI, pulmonary artery systolic pressure (PAS), IVRT/T _E-e′_ and Ar-A [15].

**Table 1:** Demographics and clinical characteristics of the study population

**Participants (n=696)**	**Age 20–40 years (n, %)**	**Age 41–60 years (n, %)**	**Age 61–80 years (n, %)**	**Male (n, %)**
**Healthy subjects** (n=100)	32 (32%)	35 (35%)	33 (33%)	50 (50%)
**HTN pts.** (n=299)	96 (32.1%)	105 (35.1%)	98 (32.7%)	148 (49.4%)
HTN mild (n=100)	33 (33%)	34 (34%)	33 (33%)	48 (48%)
HTN moderate (n=100)	31 (31%)	35 (35%)	34 (34%)	53 (53%)
HTN severe (n=99)	32 (31.68%)	36 (35.64%)	31 (30.69%)	47 (46.53%)
**DD pts.** (n=297)	95 (31.9%)	99 (33.3%)	103 (34.6%)	153 (51.5%)
DD impaired relax. (n=101)	32 (31.7%)	34 (33.7%)	35 (34.6%)	47 (46.5%)
DD pseudonormal. (n=99)	32 (32.3%)	33 (33.3%)	34 (34.3%)	52 (52.5%)
DD restriction (n=97)	31 (32%)	32 (33%)	34 (35%)	54 (55.7%)
Subjects in the groups (n, %)	223 (32%)	239 (34.3%)	234 (33.6%)	352 (50.6%)

**Table 2:** Demographics and clinical characteristics of the study population

**Participants (n=696)**	**Healthy subjects (n=100)**	**HTN pts. (n=299)**	**DD pts. (n=297)**	**p-value**
Height (cm)	170±14	168±16	168±14	0.241
Weight (kg)	66±7	67±9	65±9	0.311
BMI (kg/m^2^)	22.9±2	23.3±2	23.1±2	0.233
Heart rate (bpm)	70±10	72±10	68±10	0.08
Systolic blood pressure (mmHg)	125±5	128±5	130±5	<0.0001
Diastolic blood pressure (mmHg)	70±8	78±8	80±8	<0.0001
EF (Simpson’s method %)	64.4±2%	63.7±4%	63.9±4%	0.161

**Figure 1:**
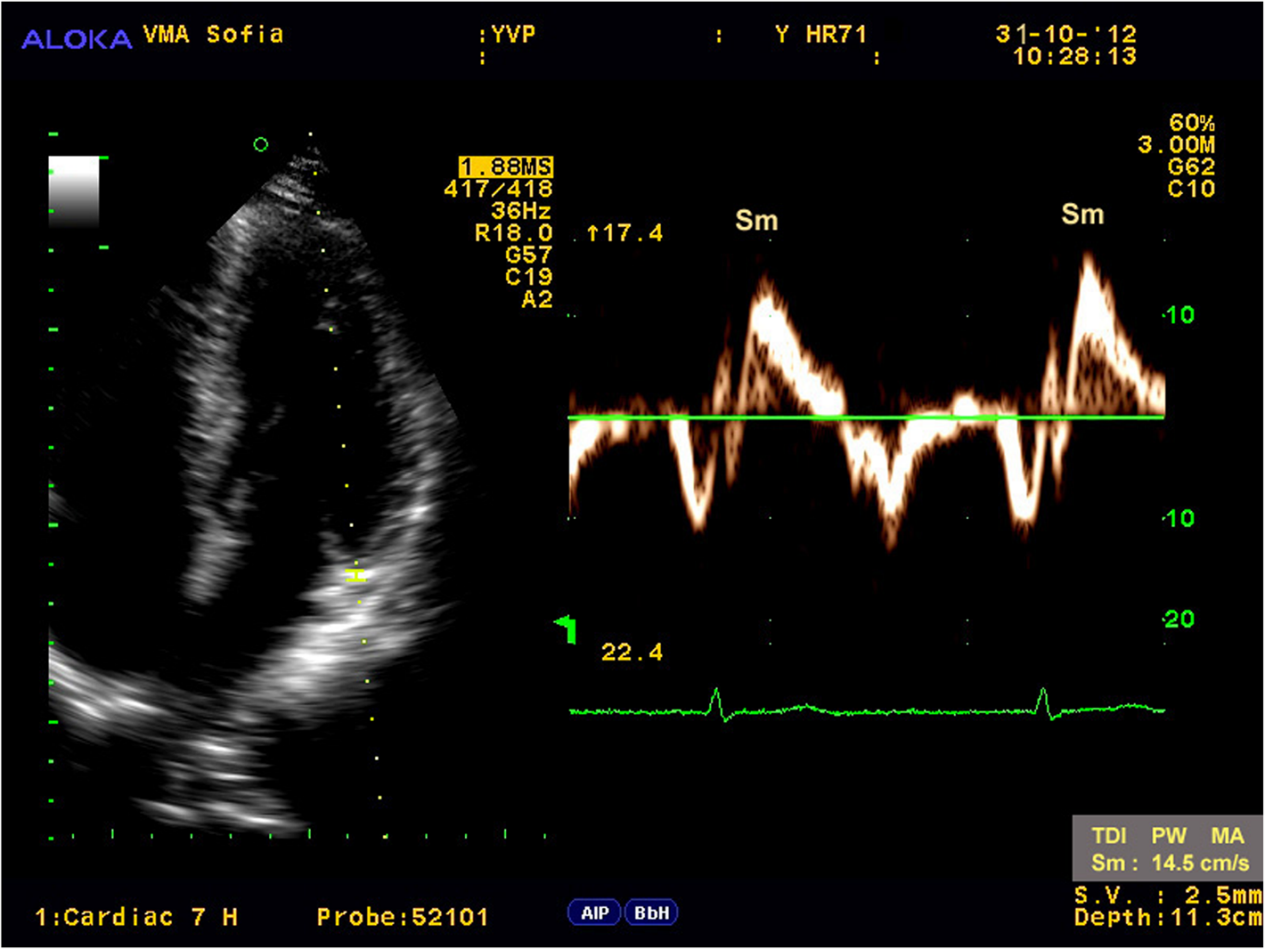
Peak Sm velocity obtained from the lateral site of the mitral annulus using spectral PW TDI.

**Figure 2:**
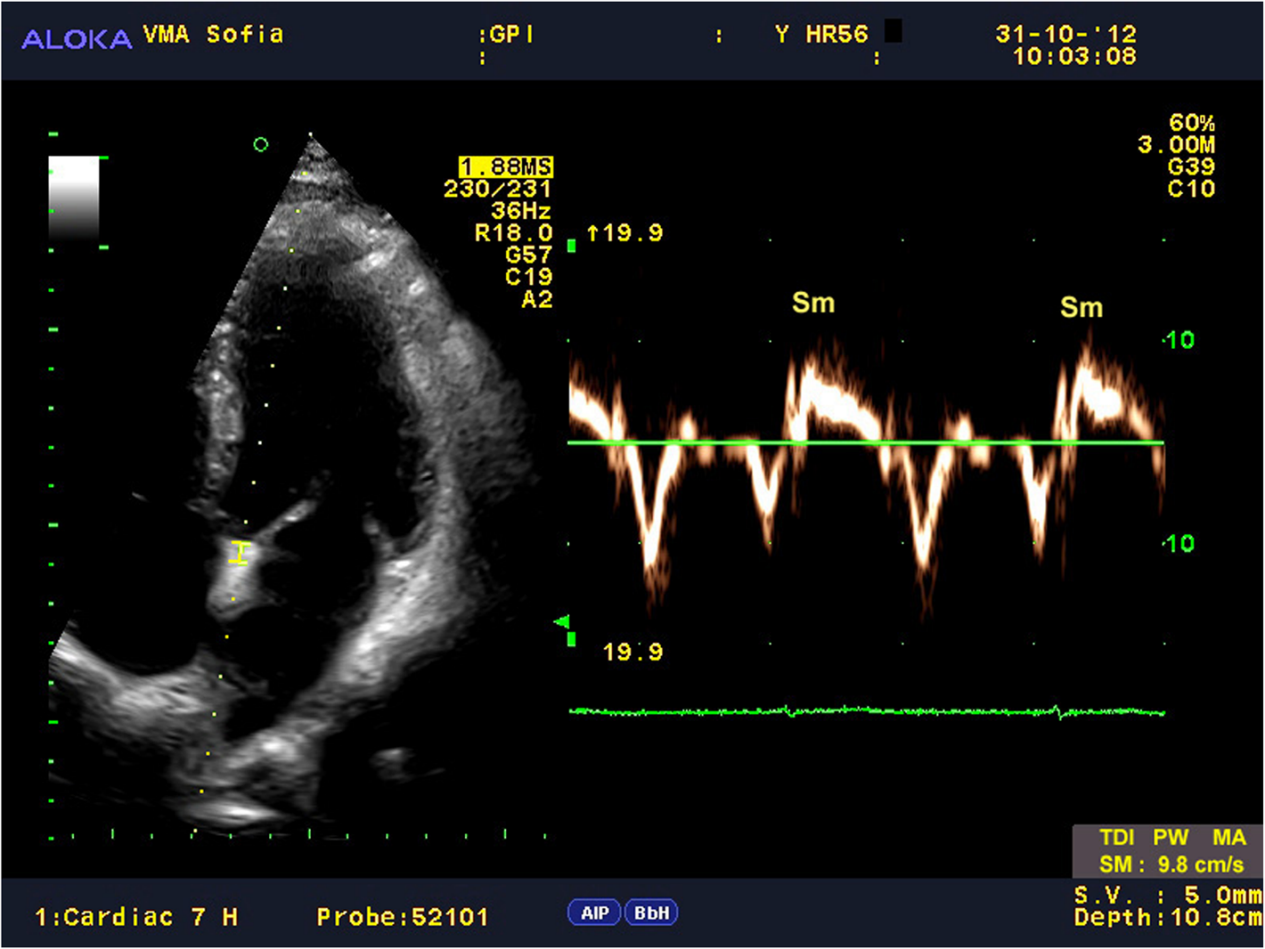
Peak Sm velocity obtained from the septal site of the mitral annulus using spectral PW TDI.

Myocardial velocities were measured on-line using spectral pulse wave TDI. The sample volume was acquired using low velocity, high-intensity myocardial signals at a high frame rate (>150 MHz). The imaging angle was adjusted to ensure as near to a parallel alignment of the beam as possible with the myocardial segment of interest. The longitudinal contraction of the LV was investigated by the average peak systolic velocity of the mitral annulus (Sm_avg_) using two positions, septal and lateral, from the apical 4-chamber view (Figure 1, Figure 2). In accordance with the study protocol, 3 consecutive complexes were analyzed, and the mean value was calculated. After each evaluation, the results were processed with the АLOKA D4D software and digitally stored (post-processing) to evaluate the reliability of the results. Two experienced echocardiographers evaluated the results of 10 randomly selected participants from every group, independently from one another, for the analysis of intra- and inter-observer variability.

### Statistical analysis

The statistical analysis was performed using SPSS (Statistical Package for Social Science) version 15.0 for Windows. The significance level for this study was set at *p* < 0.05. Categorical data are shown by frequencies and percentages. To determine the strength of the correlation between the variables, we used the Pearson’s r correlation coefficient. Continuous variables and their association with age and gender were assessed using repeated measures ANOVA and multiple regression analysis. References ranges are presented as values denoting the 5^th^ and 95^th^ percentiles. We performed the analysis in subgroups using an unpaired *t*-test. Inter- and intra-observer variability of the Sm_(avg)_ and the component variables were assessed using a coefficient of variances (CV) according to the following formula [16]:

$$\begin{array}{cc} \;\text{CV}= & \left[\left(\text{measurment}1-\text{measurment}2\right)\right)/\\ & \left(\text{arithmetic mean of measurement}\right]\times 100 \end{array}$$

Systematic bias between repeated measurements was assessed using a Bland-Altman analysis [17].

To assess the reliability of the measurements we used intraclass correlation coefficient (ICC).

## Results

The main goal of the statistical analysis was to investigate the significance of the variances of Sm_(avg)_ in the observes groups. We used repeated measures ANOVA and multiple regressions.

ANOVA generates 3 tests. The first one (sphericity) refers to the differences between serial measurements of Sm_(avg)_. The results showed that the variances of velocities are reliable to investigate in the whole study population. The second test (between subjects effects) refers to the variations in Sm_(avg)_ when the source are age and gender. According to the results, we can state that age and gender significantly influent on the velocities variations. The third test (within subject’s effects) precisely presents the interactions of HTN and DD with the age and gender. We found that the variances of velocities mainly depend on the group membership of the subjects. It means that HTN and DD are independents factors whose influence superimposes those of the age and gender (Table 3).

**Table 3:** Statistical analysis

**Repeated measures ANOVA Sample size (n=696)**			**Multiple regressions Sample size (n=696)**		
	Source of variation	*P*	*Dependent variable*	*R*	*P*
			(*Age 20*–*40*,*41*-*60*,*up to 61*)		
			*Independents Variables* (*Gender*/*HTN*/*DD*)		
*Sphericity*	Huynh-Feldt (0,326)		Multiple r	=0,915	<0,0001
	Greenhouse-Geisser (0,324)	<0,0001	Healthy male	−0,838	<0,0001
			Healthy female	−0,821	<0,0001
*Test of Between*-*Subjects Effects*	Age and Gender	<0,0001	HTN male	−0,734	<0,0001
			HTN female	−0,580	<0,0001
*Test of Within*-*Subjects Effects*	Age and Gender	<0,0001	DD male	−0,891	<0,0001
	HTN and DD		DD female	−0,882	<0,0001

The multiple regressions showed a strong negative correlation between aging and the longitudinal contraction of LV. It can be concluded that aging is a strong factor, significantly related to the decrease of Sm_(avg)_ in every group. We found a higher Sm_(avg)_ in males compared with females, as well as a step-wise decrease of Sm_(avg)_ with aging (Table 4).

**Table 4:** **Limits of Sm**_**(avg) **_**in Healthy pts. (n=100)**

**Gender/Age**	**20-40 years**	**41-60 years**	**61-80 years**	**p-value**	**Limits**
*Male* (*n*=*50*)	>10.4±0.1 (cm/s)	10.4-10.3±0.1 (cm/s)	10.3±0.1(cm/s)	*0*.*01*	*10*.*4*-*10*.*2* (cm/s)
*Female* (*n*=*50*)	>10.3±0.1 (cm/s)	10.3-10.2±0.1 (cm/s)	10.2±0.1(cm/s)	*0*.*01*	
	*p* = *0*.*01*	*p* =*0*.*01*	*p* =*0*.*01*		

Analysis showed significant differences in Sm_(avg)_ between controls and patients with HTN. In subjects with HTN without DD, there was a significant step-wise reduction in Sm_(avg)_ for every grade of HTN (Table 5). Similarly, in patients with HTN and DD, velocity was substantially lower for every grade of DD (Table 6). The data showed limits between different groups (normal subjects, HTN, and DD), which are well stratified by velocity. According to the analysis, the control group had Sm_(avg)_ of more than 10.2 cm/s. The patients with HTN without DD demonstrate Sm_(avg)_ in the boundaries of 10.2-9.9 cm/s when considering gender and level of HTN. That is, younger males with mild HTN, will have higher velocities compared with older females with moderate or severe HTN who will have lower velocities. In patients with already developed DD, Sm_(avg)_ falls below 9.9 cm/s. Our data show that the limit is below 9.8 cm/s when considering gender and the magnitude of DD. This means that younger, male patients with DD-impaired relaxation will be situated in the upper end of this limit in contrast to older females who have pseudonormalization or restriction, who will be positioned in the lower end of the boundary.

**Table 5:** **Limits of Sm**_**(avg) **_**in HTN pts. (n=299)**

**Gender**	**Grade**	**Mild HTN (n=100)**	**Moderate HTN (n=100)**	**Severe HTN (n=99)**	**p-value**	**Limits**
*Male* (*n*=*148*)		10.2±0.1 (cm/s)	10.2-10.1±0.1 (cm/s)	10.1±0.1 (cm/s)	<*0*.*0001*	*10*.*2*-*9*.*9* (cm/s)
*Female* (*n*= *151*)		10.1±0.1 (cm/s)	10.1-9.9±0.1 (cm/s)	9.9±0.1 (cm/s)	<*0*.*0001*	

**Table 6:** **Limits of Sm**_**(avg) **_**in DD pts. (n=297)**

**Gender**	**Grade**	**Impaired relaxation (n=101)**	**Pseudonormalization (n=99)**	**Restriction (n=97)**	**p-value**	**Limits**
*Male* (*n*=*153*)		9.8±0.1 (cm/s)	9.5±0.1 (cm/s)	9.3±0.01 (cm/s)	<*0*.*0001*	*9*.*8*-*8*.*8* (cm/s)
*Female* (*n*=*144*)		9.7±0.01 (cm/s)	9.2±0.01 (cm/s)	8.8±0.01 (cm/s)	<*0*.*0001*	

**Figure 3:**
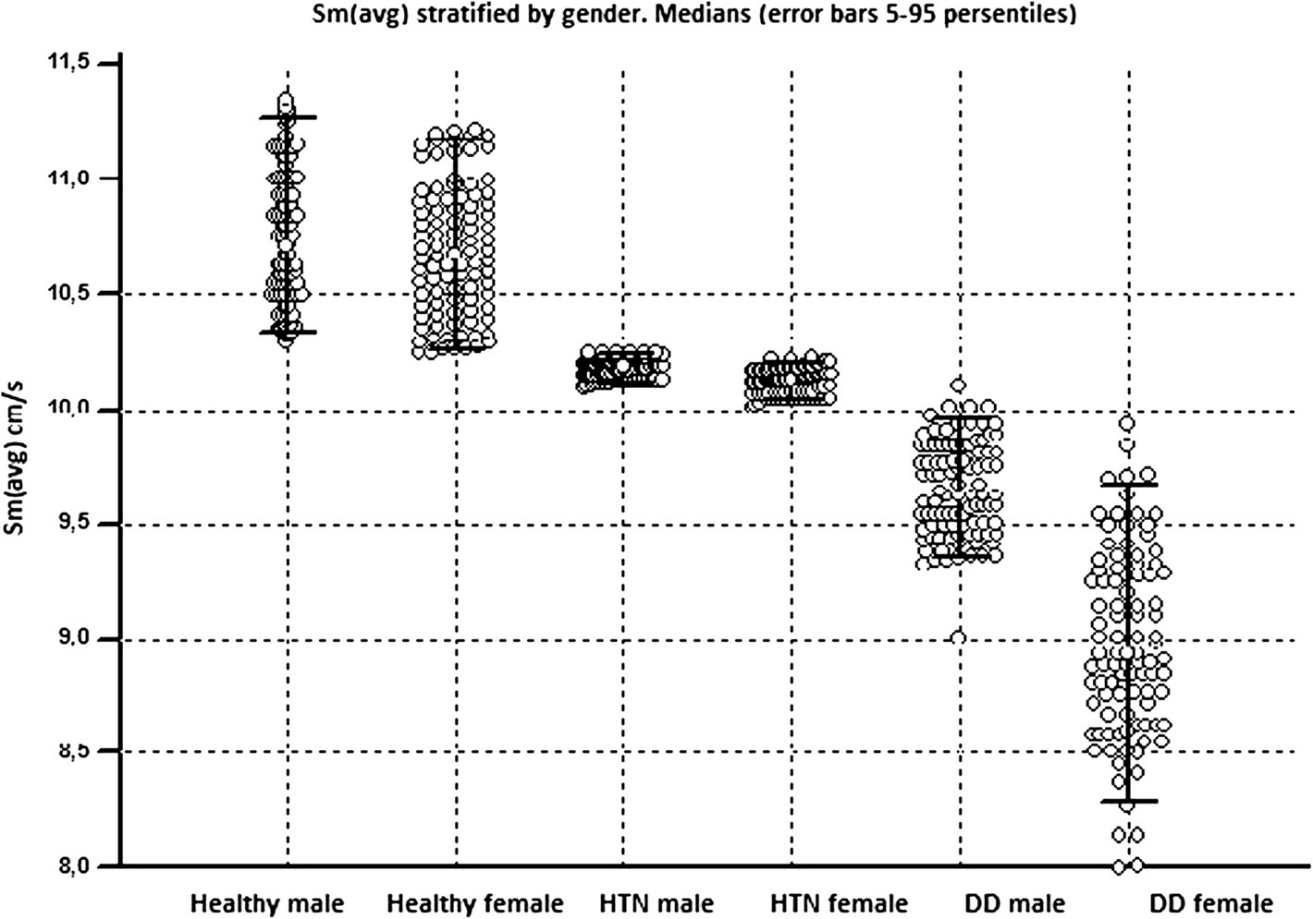
Averaged mitral Sm stratified by gender.

The underlying explanation of the above scheme is that there is age and gender-dependent changes in velocity superimpose those due to HTN and DD (Figure 3). According to our data, if Sm_(avg)_ is less than 10.2 cm/s the probability of developing early longitudinal systolic dysfunction as a result of HTN is higher. Likewise, if Sm_(avg)_ is less than 9.8 cm/s, the probability of developing DD due to HTN is also higher. This is a simple two-cutoff approach for detection of the early changes in LV longitudinal systolic function using Sm_(avg)_.

### Reproducibility of the Sm_(avg)_

To assess the reliability of the measurements we used intraclass correlation coefficient (ICC). The results are summarized in Table 7. Inter- and intra-observer variability of the Sm_(avg)_ and the component variables were assessed using a coefficient of variances (CV). Systematic bias between repeated measurements was assessed using a Bland-Altman analysis. The mean values for Sm_(avg)_ and the components variables, the results of the Bland-Altman analysis, and the CV for both inter- and intra-observer variability are summarized in Table 8. The absolute change of the intra and inter observer variability is between 8 and 13%.

**Table 7:** Intraclass correlation coefficient (ICC)

**Participants (n=210)**	**ICC (1 observer)**	**95% CI**	**Strength of agreement**	**ICC (2 observers)**	**95% CI**	**Strength of agreement**
**Healthy** (n=30)	*Single measures*			*Average measures*		
Sm _lat._ (cm/s)	0.7358	0.5329 ÷ 0.8529	Good	0.8478	0.6953 ÷ 0.9240	Very good
Sm _sep._ (cm/s)	0.9911	0.9867 ÷ 0.9941	Very good	0.9955	0.9933 ÷ 0.9970	Very good
Sm _(avg)_ (cm/s)	0.8634	0.8010 ÷ 0.9010	Very good	0.9216	0.8901 ÷ 0.9450	Very good
**HTN** (n=90)						
Sm _lat._ (cm/s)	0.7338	0.5170 ÷ 0.8622	Good	0.8465	0.6816 ÷ 0.9260	Very good
Sm _sep._ (cm/s)	0.9337	0.9020 ÷ 0.9553	Very Good	0.9653	0.9485 ÷ 0.9772	Very good
Sm _(avg)_ (cm/s)	0.8337	0.8015 ÷0.8080	Very Good	0.9059	0.8095 ÷ 0.9210	Very good
**DD** (n=90)						
Sm _lat._ (cm/s)	0.7841	0.6924 ÷ 0.8509	Good	0.8790	0.8183 ÷ 0.9194	Very good
Sm _sep._ (cm/s)	0.8049	0.6338 ÷ 0.9009	Very Good	0.8919	0.7759 ÷ 0.9479	Good
Sm _(avg)_ (cm/s)	0.7945	0.7234 ÷ 0.8222	Good	0.8854	0.8005 ÷ 0.9201	Very good

The ICC is a measure of the reliability of measurements or ratings. The table reports two coefficients with their respective 95% CI. Single measures: this is an index for the reliability of the ratings for one, typical, single rater and average measures which is an index for the reliability of different raters averaged together. (<0.20 Poor; 0.21-0.40 Fair; 0.41-0.60 Moderate; 0.61-0.80 Good; 0.81-1.00 Very good).

**Table 8:** **Inter- and intra-observer variability of the Sm**_**(avg)**_

	**Intra-observer**			**Inter-observer**		
**Participants (n=210)**	**Mean±SD (1 observer)**	**Bland-Altman (95% CI)**	**CV%**	**Mean±SD (2 observers)**	**Bland-Altman (95% CI)**	**CV%, pooled mean±SD**
**Healthy male/female** (n=30)						
Sm _lat._ (cm/s)	10.2±0.1	0.7 (−1.0 ÷ 2.0)	**12.5**	10.3±0.1	2.5 (−3.5 ÷ 1.5)	**13.0 ± 1.0**
Sm _sep._ (cm/s)	10.4±0.1	1.13(−1.5 ÷ 1.5)	**8.5**	10.4±0.1	2.3 (−0.2 ÷ 2.4)	**9.0 ± 1.0**
Sm _(avg)_ (cm/s)	10.3±0.1	1.5 (−1.0 ÷ 1.95)	**9.0**	10.4±0.1	2.2 (0.1 ÷ 2.5)	**10.0 ± 1.0**
**HTN male/female** (n=90)						
Sm _lat._ (cm/s)	10.1±0.1	0.5 (−2.2 ÷ 2.2)	**10.5**	10.1±0.1	0.3 (0.0 ÷ 0.5)	**12.0 ± 1.0**
Sm _sep._ (cm/s)	10.2±0.1	1.2 (−1.2 ÷ 1.2)	**7.5**	10.2±0.1	−2.8 (−3.0 ÷ −0.5)	**8.0 ± 1.0**
Sm _(avg)_ (cm/s)	10.1±0.1	−2.5 (−3.0 ÷1.0)	**9.5**	10.1±0.1	1.4(−1.1 ÷ 2.8)	**10.5 ± 1.0**
**DD male/female** (n=90)						
Sm _lat._ (cm/s)	9.2±0.1	1.0 (−1.5 ÷ 1.5)	**11.5**	8.9±0.1	0.95 (−0.2 ÷ 1.0)	**12.5 ± 1.0**
Sm _sep._ (cm/s)	9.5±0.1	0.25 (−1.5 ÷ 1.5)	**8.0**	9.4±0.1	1.13 (−1.5 ÷ 1.5)	**10.0 ± 1.0**
Sm _(avg)_ (cm/s)	9.3±0.1	−2.7 (−3.0 ÷ −1.0)	**9.0**	9.2±0.1	−1.8 (−2.4 ÷ 0.6)	**10.0 ± 1.0**

**CV** = [(measurment1 − measurment2)/arithmetic mean of measurement] × 100.

Analyzing the results from Tables 7 and 8 we can state that the Sm of the septal site of the mitral annulus shows better reliability than the lateral site. The Sm_(avg)_, demonstrates better reproducibility than the lateral site alone. Summarizing the above results, we can conclude that the peak systolic velocity from the septal site shows the best reliability. This result could be explained by the beneficial orientation of the septal position, which is parallel to the Doppler beam, and the angle is close to zero. This means that the expected error during the investigation should be negligible. The lateral site is not parallel to the Doppler beam, and the angle which closes is greater than zero, hence the probability of error is higher. The Sm_(avg)_ summarizes the advantages and disadvantages of the Sm from both mitral sites, which is why it should be used in clinical practice.

### Comparison with previous studies

In studies conducted previously, there was little information regarding the influence of age and gender on the longitudinal contraction of LV. Most researchers support the hypothesis that the longitudinal systolic function reduces with aging [18-20]. Such a relationship was recently demonstrated again in the Copenhagen City Heart Study [4]. After the age of 25, the velocity’s curve starts to move slowly away from normal values and shift to lower velocities in the elderly. The investigators reported higher systolic velocities in men and lower systolic velocities in women.

Several studies have attempted to derive normative values for TDI parameters, but they have tended to explore the association of these parameters with age and have consequently reported only their mean values [21-23]. The first percentile-based study with regard to both systolic and diastolic TDI parameters was provided by Chahal et al [24]. The investigators defined a large reference limit for Sm_(avg)_ for normal subjects aged 35–75 years (6,8-12,2 cm/s). An important difference between our and Chahal’s study is the lower border of velocity in healthy older subjects. According to our results, additional heart pathology should be added to gender and aging, such as a combination of HTN and DD, for the velocity to be reduced to such a low level. In our opinion, gender and aging alone cannot lead to this reduction.

Three studies have shown that patients with HTN have lower systolic and early diastolic velocities in the long axis compared with healthy subjects [4-6]. Recently, data from the Copenhagen City Heart Study confirmed these findings. In our study, we observed a tendency for a reduction in the longitudinal contraction of the LV, which starts early in the course of HTN, with minimal changes in systolic long axis function. Later, with the progression of the disease, in a step-wise manner and simultaneously with the grade of HTN, a larger reduction in systolic long axis function was found.

Six studies support the hypothesis that patients with DD have a mild impairment in the longitudinal systolic function of the LV [7,8]. The data of our study do not add new information to existing. But we would suggest that this impairment is a step before EF begins to drop in spite of the fact that this requires serial measures of the EF respectively of the Sm. The protocol of our study does not provide serial evaluation of the EF below 45% which is the one of the major limitation.

The main differences between all these trials and our study are the methods of investigation, the number of participants and the design.

## Discussion

**Figure 4:**
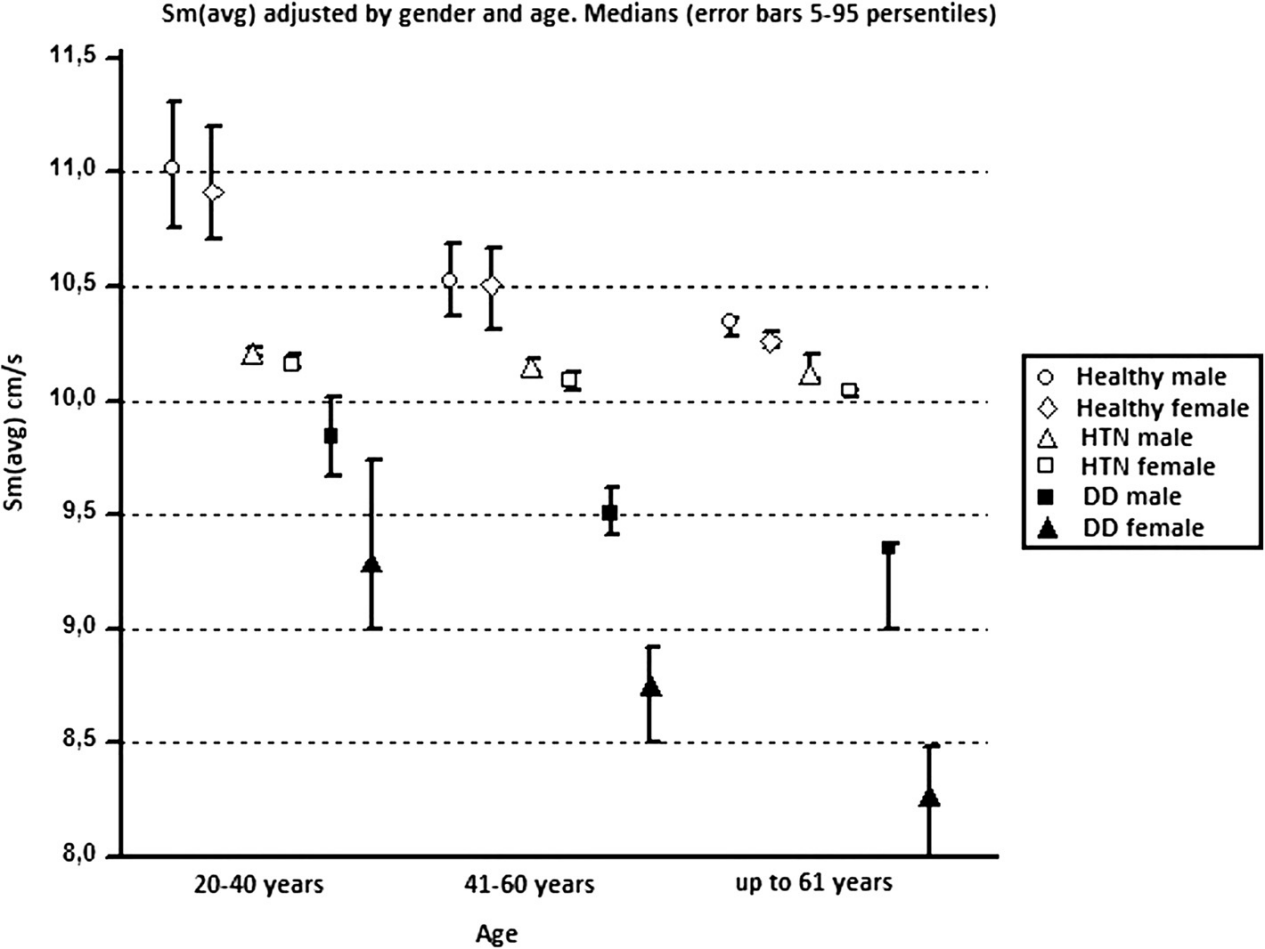
Averaged mitral Sm adjusted by age and gender.

Although our study is not innovative in nature, as it repeats some of the conclusions achieved more than ten years ago, it confirms the hypothesis that in its development, HTN leads to early changes in the global systolic function of the LV. We concluded that the patients with HTN develop a step-wise reduction in the global systolic function, which is age and gender dependent, and more importantly, that this happens before the onset of DD. The question is how to avoid reaching this stage, and how to diagnose the patient at risk of developing heart failure with preserved and later reduced EF. The general conclusion of the study regards the existence of three trends for the reduction of the long axis systolic function. The first trend is one of age, the second depends on gender and the third concerns the interdependency of HTN and DD. All three trends interact in such a way that the estimated Sm_(avg)_ is a reflection of the gender, age and grade of HTN and the magnitude of DD of the corresponding subject (Figure 4).

In summary, our results show that HTN and its evolution are related to a slight reduction in the longitudinal systolic contraction of the LV, and the subsequent DD adds further decline to the already existing mild systolic dysfunction. This finding alone cannot account for the clinical symptoms in patients, such as breathlessness and fatigue, for example. In most cases, these symptoms occur as a result of elevated LV filling pressure. Therefore the importance of our results lies in the fact that these initial changes in systolic contraction of the LV could be used as an early sign that should prompt optimization of the treatment of HTN. The strength of the study is the analysis of incremental changes in longitudinal contraction but not so many the classification of the degree of systolic dysfunction. It is critically important to discover this initial contractile dysfunction when the EF is still preserved. The aim of treatment is to prevent the further development of DD and, thus, heart failure. It is reasonably to plane and conduct a prospective study which elucidate whether the different therapeutic strategy could influent to this initial preclinical systolic dysfunction. We could suggest benefits following this hypothesis, but at this point of view couldn’t prove it.

Finally, we should emphasize that the investigation of early changes in the longitudinal systolic function of the LV is always experience dependent but not time-consuming, and all cardiologists should be trained in it.

### Limitations

Our study is limited by the fact that all participants were free of comorbidities. Typically, in clinical practice we see a combination of HTN with coronary heart disease, diabetes mellitus, chronic kidney diseases, anemia, etc. It has been proven that the above-mentioned conditions increase cardiovascular mortality or worsen the course of heart disease, especially when DD is present. Further investigation involving participants with various comorbidities is warranted to investigate the role of each disease in the reduction of longitudinal contractility of the LV.

## Conclusions

The results of this study show that HTN and its evolution are related to slight impairments of the longitudinal systolic function. Without a prompt change in therapy the next stage is worsens the global contractility due to further progression to DD. The main objective should be to diagnose this preclinical systolic dysfunction early in the course of the HTN before there are morphological changes in the LV. Adjustments to therapy should be initiated before DD develops.

## Competing interest

The authors declare that they have no competing interests

## Authors’ contributions

ID, PP, and LD planned the study, investigated all patients, performed measurements and analyzed the data. ID and LD performed statistical analysis and wrote the manuscript. ID and PP made critical review of the paper. All authors read and approved the final manuscript.
